# Immobilization of lipid nanorods onto two-dimensional crystals of protein tamavidin 2 for high-speed atomic force microscopy

**DOI:** 10.1016/j.xpro.2023.102633

**Published:** 2023-12-02

**Authors:** Daisuke Noshiro, Nobuo N. Noda

**Affiliations:** 1 Institute for Genetic Medicine, Hokkaido University, Sapporo, Hokkaido 060-0815, Japan; 2 Institute of Microbial Chemistry (BIKAKEN), Shinagawa-ku, Tokyo 141-0021, Japan

**Keywords:** Atomic Force Microscopy, AFM, Biotechnology and Bioengineering, Protein Expression and Purification

## Abstract

High-speed atomic force microscopy is a technique that allows real-time observation of biomolecules and biological phenomena reconstituted on a substrate. Here, we present a protocol for immobilizing lipid nanorods onto two-dimensional crystals of biotin-binding protein tamavidin 2. We describe steps for the preparation of tamavidin 2 protein, lipid nanorods, and two-dimensional crystals of tamavidin 2 formed on mica. Immobilized lipid nanorods are one of the useful tools for observation of specific proteins in action.

For complete details on the use and execution of this protocol, please refer to Fukuda et al. (2023).^1^

## Highlights

• Lipid nanorod immobilization onto tamavidin 2 two-dimensional crystals for high-speed AFM
• Steps for preparing tamavidin 2 protein, 2D crystals, and lipid nanorods
• High-speed AFM imaging of 2D crystals of tamavidin 2 and immobilized lipid nanorods

## Abstract (editor highlights)

Publisher’s note: Undertaking any experimental protocol requires adherence to local institutional guidelines for laboratory safety and ethics.

## Graphical abstract

**Figure:**
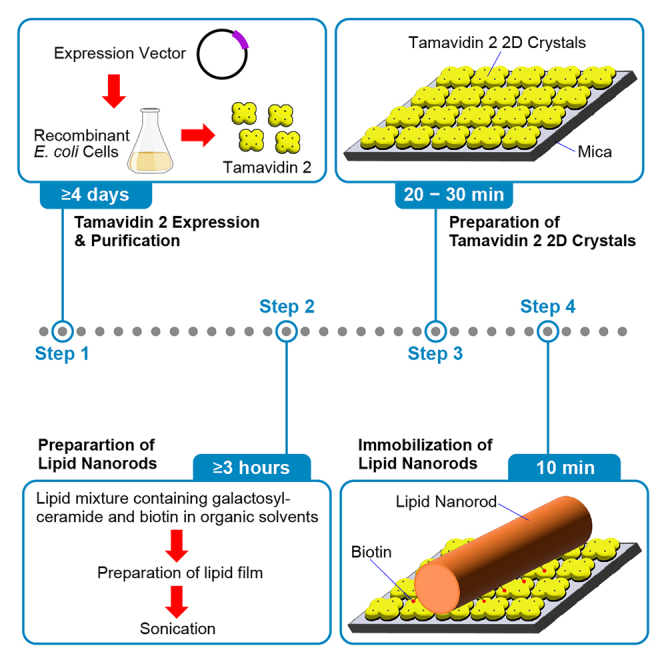

## Before you begin

**Figure 1:**
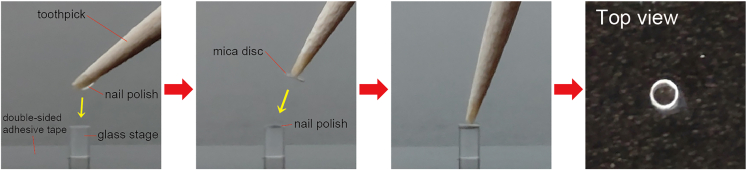
The method to glue a mica disc on a glass stage It is recommended to perform these under a stereo microscope.

This protocol describes a method to prepare lipid nanorods (30–50 nm in diameter) on a substrate for high-speed atomic force microscopy (HS-AFM) observations. Lipid mixtures containing galactocerebrosides spontaneously form nanorods. In this protocol, biotin-containing lipid nanorods are immobilized on two-dimensional (2D) crystals of biotin-binding protein tamavidin 2. Tamavidin 2 2D crystals can be formed directly on mica surface. We use mica disc (1.5 mm in diameter) glued on the top of a glass stage (1.5 mm in diameter and 2.0 mm in height). This section describes our modified method from the protocol by Uchihashi et al.^2^ and the manual provided by Research Institute of Biomolecule Metrology Co., Ltd. (Ibaraki, Japan) to prepare mica glued on the glass stage (Figure 1).

### Prepare mica glued on a glass stage

It is recommended to perform these operations under a stereo microscope.

1. Set a glass stage on double-sided adhesive tape.
2. Put a small amount of nail polish on the tip of a toothpick (or a micropipette tip (0.1–10 μL)).

***Note:*** Epoxy adhesives can be used instead of nail polish.^2^

3. Apply and spread nail polish on the top of the glass stage.
4. Touch and pick up a mica disc using the toothpick with very little nail polish.
5. Put the mica disc on the top of the glass stage and move the mica disc to the center.
6. Wait ≥3 h for drying.

***Note:*** The mica disc can be removed using acetone.

## Key resources table

**Table:** 

REAGENT or RESOURCE	SOURCE	IDENTIFIER
**Chemicals, peptides, and recombinant proteins**		

pET-17b-tamavidin 2	GenScript	N/A
Ampicillin sodium salt (25 g)	Nacalai Tesque	19769-22
Isopropyl-β-D-thiogalactopyranoside (100 g)	Nacalai Tesque	19742-07
Phenylmethylsulfonyl fluoride (25 g)	Nacalai Tesque	27327-52
Dimethyl sulfoxide (500 g)	Nacalai Tesque	13407-45
NaCl (500 g)	Nacalai Tesque	31320-05
CAPS (100 g)	FUJIFILM Wako	343-00484
Ammonium acetate (500 g)	Nacalai Tesque	02406-95
Potassium dihydrogen phosphate (500 g)	FUJIFILM Wako	169-04245
Dipotassium hydrogenphosphate (500 g)	FUJIFILM Wako	164-04295
Iminobiotin agarose (5 mL)	Thermo Fisher Scientific	20221
HEPES (500 g)	Nacalai Tesque	17514-15
Magnesium chloride hexahydrate (500 g)	Nacalai Tesque	20909-55
Polyethylene glycol 6,000 (molecular weight: 7,300–9,300) (500 g)	FUJIFILM Wako	169-22945
25% Glutaraldehyde solution (25 mL)	FUJIFILM Wako	079-00533
C24:1 galactosyl(β) ceramide (5 mg)	Avanti Polar Lipids	860546P
18:1 Biotinyl Cap PE (25 mg)	Avanti Polar Lipids	870273P

**Software and algorithms**		

Kodec4.4.7.39	N/A	https://elifesciences.org/articles/04806

**Other**		

Mica substrate (φ1.5 mm)	Research Institute of Biomolecule Metrology	N/A
Glass stage (φ1.5 × t2.0 mm)	Research Institute of Biomolecule Metrology	N/A
Integrate top and base coat N (4 mL)	Shiseido	N/A
Bioshaker G·BR-200	Taitec	0058178-000
High-speed refrigerated centrifuge model 7000	KUBOTA	N/A
Benchtop pH meter	AS ONE (HORIBA)	65-0509-31
Stericup quick release-GV sterile vacuum filtration system (1000 mL process volume, 0.22 μm pore size)	Millipore	S2GVU11RE
Econofltr (0.2 μm pore size)	Agilent Technologies	5190–5275
Branson Sonifier 250	N/A	N/A
Econo-Pac chromatography columns (20 mL)	Bio-Rad	732-1010
Amicon Ultra-15 centrifugal filter units (10 kDa NMWL)	Merck Millipore	UFC801024
NanoDrop 2000c spectrophotometer	Thermo Fisher Scientific	ND-2000C
LABORAN sample tube bottle 5 mL	AS ONE	9-851-04
Ultrasonic cleaner (237 × 235 × 290 mm)	AS ONE	1-2160-01
Ultrasonic dispersion machine (182 × 320 × 110 mm)	AS ONE (SMT Corporation)	5-4030-01
Asnol Petri dish JP (φ90 × 15 mm)	AS ONE	3-1491-51
Sample scanning HS-AFM instrument (MS-NEX)	Research Institute of Biomolecule Metrology	N/A
Ultra-short cantilevers (for high-speed AFM)	NanoWorld	USC-F1.2-k0.15

## Materials and equipment

### Ampicillin (100 mg/mL)

Add deionized distilled (ddH_2_O) to 1.00 g of ampicillin up to 10 mL (total volume). Vortex until ampicillin is completely dissolved. Filter the solution through a 0.2-μm syringe filter. Dispense the solution into 1.0-mL aliquots. Ampicillin (100 mg/mL) can be stored at −20°C for up to 1 year.

### Isopropyl-β-D-thiogalactopyranoside (IPTG) (0.5 M)

Add ddH_2_O to 1.19 g of IPTG up to 10 mL (total volume). Vortex until IPTG is completely dissolved. Filter the solution through a 0.2-μm syringe filter. Dispense the solution into 1.0-mL aliquots. IPTG (0.5 M) can be stored at −20°C for up to 1 year.

### Phenylmethylsulfonyl fluoride (PMSF) (1 M)

Add dimethyl sulfoxide (DMSO) to 1.74 g of PMSF up to 10 mL (total volume). Vortex until PMSF is completely dissolved. Dispense the solution into 50-μL aliquots. PMSF (1 M) can be stored at −20°C for up to 1 year.

### NaCl (1 M)

Dissolve 58.4 g of NaCl in ∼700 mL of ddH_2_O. Add ddH_2_O up to 1000 mL. Filter the solution through a 0.22-μm filter membrane. NaCl (1 M) can be stored at ∼23°C for up to 1 year.

### N-cyclohexyl-3-aminopropanesulfonic acid (CAPS), pH 11.0 (0.2 M)

Dissolve 4.43 g of CAPS in ∼70 mL of ddH_2_O and adjust pH to 11.0 with 1 N of NaOH (∼13.2 mL). Add ddH_2_O up to 100 mL. Filter the solution through a 0.22-μm filter membrane. CAPS (0.2 M, pH 11.0) can be stored at ∼23°C for up to 1 year.

**Table:** Buffer L (50 mM of CAPS-NaOH containing 50 mM of NaCl, pH 11.0)

Reagent	Final concentration	Amount
NaCl (1 M)	50 mM	5 mL
CAPS-NaOH pH 11.0 (0.2 M)	50 mM	25 mL
ddH_2_O	N/A	up to 100 mL
**Total**	**N/A**	**100 mL**

Filter the mixed solution through a 0.22-μm filter membrane. Buffer L can be stored at 4°C for up to 1 year.

**Table:** Buffer W (50 mM of CAPS-NaOH containing 500 mM of NaCl, pH 11.0)

Reagent	Final concentration	Amount
NaCl (1 M)	500 mM	100 mL
CAPS-NaOH, pH 11.0 (0.2 M)	50 mM	50 mL
ddH_2_O	N/A	up to 200 mL
**Total**	**N/A**	**200 mL**

Filter the mixed solution through a 0.22-μm filter membrane. Buffer W can be stored at 4°C for up to 1 year.

### Acetic acid (3 M)

Dilute 8.57 mL of acetic acid with ddH_2_O up to 50 mL. Acetic acid (3 M) can be stored at ∼23°C for up to 1 year.

### Buffer E (50 mM of ammonium acetate, pH 4.0)

Dissolve 385 mg of ammonium acetate in ∼70 mL of ddH_2_O and adjust pH to 4.0 with 3 M of acetic acid (∼7.5 mL). Add ddH_2_O up to 100 mL. Filter the solution through a 0.22-μm filter membrane. Buffer E can be stored at 4°C for up to 1 year.

### Potassium dihydrogen phosphate (KH_2_PO_4_) (40 mM)

Dissolve 272 mg of KH_2_PO_4_ in ∼40 mL of ddH_2_O. Add ddH_2_O up to 50 mL.

### Dipotassium hydrogenphosphate (K_2_HPO_4_) (40 mM)

Dissolve 348 mg of K_2_HPO_4_ in ∼40 mL of ddH_2_O. Add ddH_2_O up to 50 mL.

### Buffer S (40 mM of potassium phosphate buffer, pH 7.0)

Add 40 mM of KH_2_PO_4_ (∼40 mL) to 50 mL of 40 mM of K_2_HPO_4_ until pH reaches 7.0. Filter the mixed solution through a 0.22-μm filter membrane. Buffer S can be stored at 4°C for up to 1 year.

### HEPES-NaOH, pH 8.5 (0.1 M)

Dissolve 2.38 g of HEPES in ∼70 mL of ddH_2_O and adjust pH to 8.5 with 1 N of NaOH (∼8.2 mL). Add ddH_2_O up to 100 mL. Filter the solution through a 0.22-μm filter membrane. HEPES-NaOH (0.1 M, pH 8.5) can be stored at ∼23°C for up to 1 year.

### MgCl_2_ (2.5 M)

Dissolve 50.8 g of MgCl_2_·6H_2_O in ∼70 mL of ddH_2_O. Add ddH_2_O up to 100 mL. Filter the solution through a 0.22-μm filter membrane. MgCl_2_ (2.5 M) can be stored at ∼23°C for up to 1 year.

**Table:** Buffer A (25 mM of HEPES-NaOH containing 500 mM of NaCl and 250 mM of MgCl_2_, pH 8.5)

Reagent	Final concentration	Amount
NaCl (1 M)	500 mM	50 mL
HEPES-NaOH, pH 8.5 (0.1 M)	25 mM	25 mL
MgCl_2_ (2.5 M)	250 mM	10 mL
ddH_2_O	N/A	up to 100 mL
**Total**	**N/A**	**100 mL**

Filter the mixed solution through a 0.22-μm filter membrane. Buffer A can be stored at ∼23°C for up to 1 year.

### Crystal buffer (20 mM of HEPES-NaOH containing 400 mM of NaCl, 200 mM of MgCl_2_ and 20% PEG6000, pH 8.5)

Add 4 mL of Buffer A to 1.0 g of PEG6000 in a 50 mL centrifuge tube. Vortex until PEG6000 is completely dissolved. The final volume will be ∼5 mL. Crystal buffer can be stored at ∼23°C for up to 1 year.

***Alternatives:*** PEG6000 (molecular weight: 7,300–9,300) may be replaced by other PEG analogs, such as PEG3350.

**Table:** Fixation buffer (20 mM of HEPES-NaOH containing 100 mM of MgCl_2_, pH 8.5)

Reagent	Final concentration	Amount
HEPES-NaOH, pH 8.5 (0.1 M)	20 mM	20 mL
MgCl_2_ (2.5 M)	100 mM	4 mL
ddH_2_O	N/A	up to 100 mL
**Total**	**N/A**	**100 mL**

Filter the mixed solution through a 0.22-μm filter membrane. Fixation buffer can be stored at ∼23°C for up to 1 year.

## Step-by-step method details

**Figure 2:**
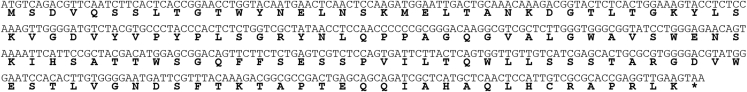
The DNA and protein sequence of tamavidin 2 from GenBank (GenBank: AB102785)

This section describes the preparation of tamavidin 2 protein, lipid nanorods and tamavidin 2 2D crystals covering the mica surface, as well as the immobilization of lipid nanorods on the 2D crystals. Tamavidin 2 is an avidin-like biotin-binding protein from *Pleurotus cornucopiae*, and it consists of 141 amino acid residues. The article by Takakura et al.^3^ contains detailed information on the discovery, properties, expression, and purification methods of tamavidin 2. The sequence of tamavidin 2 is available from GenBank (GenBank: AB102785) (Figure 2).

### Prepare the expression vector for production of tamavidin 2 protein

**Timing: 2–4 weeks**

1. Prepare an appropriate plasmid for protein expressions in *Escherichia coli* (*E. coli*) cells.

***Note:*** Expression vector pET-17b (Novagen) with the DNA sequence coding tamavidin 2 in NdeI-XhoI sites (pET-17b-tamavidin 2) in our case was designed and purchased from GenScript.

### Expression and purification of tamavidin 2 protein

**Timing: 4 days**

This subsection summarizes our modified method of expression and purification of tamavidin 2 protein from the report by Takakura et al.^3^ Tamavidin 2 is expressed at high levels in soluble fractions in recombinant *E. coli* and can be purified in a single step using a 2-iminobiotin agarose gel.

2. Transform competent *E. coli* cells (e.g., BL21(DE3)) with the expression vector.
3. Grow the *E. coli* cells harboring the expression vector in LB medium (800 mL) containing an appropriate antibiotic (e.g., 100 μg/mL of ampicillin for pET-17b vector) at 37°C until the absorbance at 600 nm reaches 0.5–0.8.
4. Add 800 μL of 0.5 M IPTG (final concentration: 0.5 mM) to the LB medium. Shake at ∼120 rpm for ∼18 h at 25°C.
5. Harvest the cells by centrifugation at 4°C (4000 × *g* for 10 min).
6. Store the cell pellet at −80°C.
7. Prepare Buffer L, Buffer W, Buffer E, and Buffer S. See the ‘materials and equipment’ section for preparation.
8. Purify tamavidin 2 following the protocol by Takakura et al.^3^ Below is the protocol with the slight modification.
  a. Resuspend the pellet in Buffer L (50 mL) and add 50 μL of 1 M phenylmethylsulfonyl fluoride (PMSF) (final concentration: 1 mM).
  b. Sonicate the suspension on ice (0.2 s of pulse with an interval of 0.8 s at ∼200 W for 10 min).
  c. Centrifuge the suspension at 4°C (18,000 × *g* for 40 min) and collect the supernatant.
  d. Purify the clear supernatant using 2-iminobiotin agarose (Thermo Fischer Scientific).
    i. Transfer 2-iminobiotin agarose resin slurry (2 mL) into an empty column (Bio-Rad).
    ii. Equilibrate the column with 4-column volumes of Buffer L.
    iii. Apply the supernatant to the column.
    iv. Wash the column with 20-column volumes of Buffer W.
    v. Elute tamavidin 2 protein by adding 5-column volumes of Buffer E.
    vi. Concentrate the eluted tamavidin 2 solution and exchange buffer to Buffer S using Amicon Ultra-15 (NMWL 10k).
    vii. Centrifuge the tamavidin 2 solution (12,000 × *g* at 4°C for 10 min) and collect the supernatant.

***Note:*** The molar extinction coefficient of tamavidin 2 is 41,750 M^−1^·cm^−1^·subunit^−1^ at 280 nm.^3^ Concentrate the tamavidin 2 solution up to 150 μM (absorbance at 280 nm = 6.26).

***Note:*** Stability of tamavidin 2 is very high and its biotin-binding activity is rarely reduced by storage in solution at 4°C for at least 1 year.^3^ In our experiment, tamavidin 2 solution purified and stored at 4°C for >3 years is used.

### Prepare lipid nanorods

**Timing: ≥3 h**

Lipid nanorods, formed by the spontaneous assembly of galactocerebrosides, have been used for HS-AFM observation of dynamin.^4^ Biotinylated lipids must be included to immobilize lipid nanorods onto tamavidin 2 2D crystals. Below is our slightly modified protocol from the report by Colom et al.^4^

9. Prepare a 100-nmol mixture of phospholipids containing galactosylceramide and biotinyl cap PE (in our case^1^: 40 μL of 1 mM galactosylceramide; 15 μL of 1 mM POPC; 5 μL of 1 mM POPE; 6 μL of 1 mM PI; 6 μL of 1 mM POPS; 6 μL of 1 mM POPA; 12 μL of 1 mM cardiolipin (CL); 10 μL of 1 mM biotinyl cap PE [40:15:5:6:6:6:12:10 mol %] in chloroform (100 μL in total)) in a 5-mL glass bottle.
10. Dry chloroform under a gentle stream of nitrogen gas to produce lipid film.
11. Place the glass bottle in a vacuum desiccator for ≥2 h to completely evaporate the chloroform.

***Note:*** You can keep the glass bottle in a vacuum desiccator for 24 h.

12. Add 100 μL of buffer you want to use (e.g., 20 mM of HEPES-NaOH containing 150 mM of NaCl, pH 7.0) to the lipid film and incubate for 10 min followed by vortexing to prepare 1 mM lipid mixture solution.
13. Transfer the lipid mixture solution to a 1.5-mL microcentrifuge tube.
14. Sonicate it in a bath sonicator for 10 min (40 W, 42 kHz).
15. Sonicate it further with a tip sonicator for ≤5 s (50 W, 20 kHz).

**CRITICAL:** Longer sonication time produces vesicular structures rather than nanorods. Details are shown in ‘problem 5’ of ‘troubleshooting’ section.

16. Protect from light and keep the 1.5-mL tube on ice until use.

### Prepare tamavidin 2 2D crystals on mica

**Timing: 20–30 min**

This subsection describes the preparation of tamavidin 2 2D crystals on mica. Tamavidin 2 2D crystals have been a useful substrate that develop directly on the mica surface to immobilize biotinylated proteins or membranes.^5^^,^^6^ See the protocol by Uchihashi et al.^2^ for HS-AFM setup and observation. We use a sample scanning HS-AFM instrument (MS-NEX, Research Institute of Biomolecule Metrology Co., Ltd.) and cantilevers (length: ∼7-μm, width: ∼2-μm, thickness: ∼0.08-μm, resonant frequency: 1.2 MHz in air, spring constant: 0.15 N/m) with electron beam deposited/EBD tips (USC-F1.2-k0.15, NanoWorld). HS-AFM images were viewed and analyzed using the software Kodec4.4.7.39.^7^

17. Prepare crystal buffer and fixation buffer. See the ‘materials and equipment’ section for preparation.
18. Add 1 μL of 150 μM of tamavidin 2 (A280 = 6.26) to 20 μL of crystal buffer (final concentration, 7.1 μM) and mix by gentle pipetting.

**CRITICAL:** Do not incubate for >60 min after the dilution with crystal buffer. Details are shown in ‘problem 2’ of ‘troubleshooting’ section.

19. Deposit a drop (∼2 μL) of the diluted tamavidin 2 solution onto freshly cleaved mica by scotch tape and incubate for 5 min.
20. Rinse the mica surface with 0.01% glutaraldehyde diluted with fixation buffer and keep the last drop on the mica for 5 min.

**CRITICAL:** Glutaraldehyde fixation after incubation of tamavidin 2 on mica is an indispensable step for stabilization of 2D crystals. Rinse with imaging buffer before glutaraldehyde fixation may wash out tamavidin 2 from the surface.

21. Rinse the mica surface with imaging buffer you want to use for HS-AFM imaging (in our case,^1^ 20 mM of HEPES-NaOH containing 150 mM of NaCl, pH of 7.0).
22. Set up HS-AFM, start imaging and confirm 2D crystals of tamavidin 2 formed on the mica surface.

**Figure 3:**
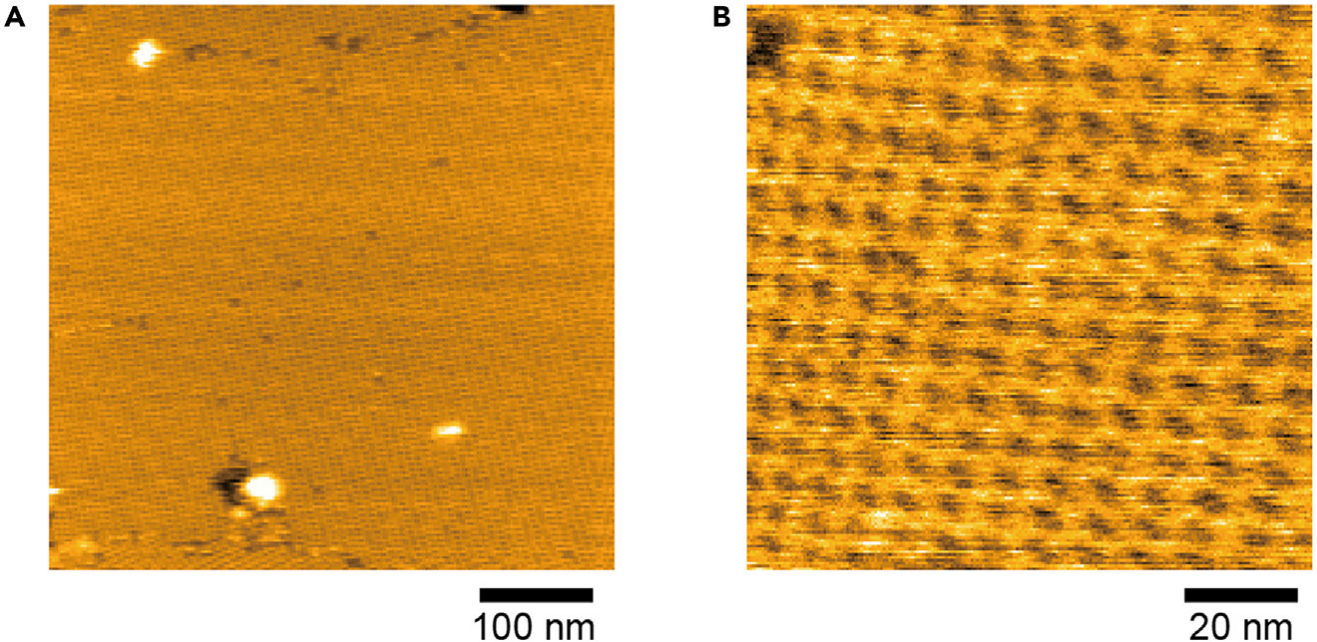
Typical HS-AFM images of tamavidin 2 2D crystals on mica surface prepared by this method (A and B) Scan range: 500 nm × 500 nm (250 × 250 pixels) (A) and 100 nm × 100 nm (200 × 200 pixels) (B); Height scale: 0–9 nm (A) and 0–1.5 nm (B). Images were acquired at 1.5 s/frame (A) and 0.6 s/frame (B).

***Note:***Figure 3 shows an example of the expected results.

23. Keep a drop of imaging buffer on the mica after HS-AFM imaging.

**Figure 4:**
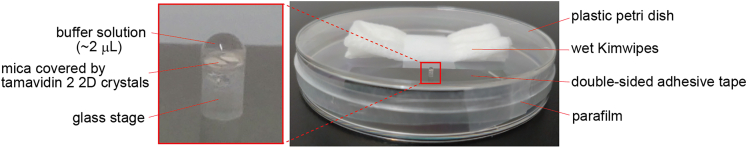
Photograph of a humid container example for keeping tamavidin 2 2D crystals on mica glued to a glass stage A sheet of Kimwipes is fixed with scotch tape and wetted with Milli-Q water in a plastic Petri dish. A drop of buffer solution (e.g., 20 mM HEPES-NaOH containing 150 mM NaCl, pH 7.0) (∼2 μL) is kept on mica. The glass stage is loosely fixed to the bottom of the Petri dish with double-sided adhesive tape to prevent it from falling over. The space between the container and the lid is sealed with parafilm.

***Note:*** The tamavidin 2 2D crystals covering the mica can be stored at ∼23°C in a humid container for several days. Figure 4 shows an example of a humid container.

### Immobilization of lipid nanorods onto tamavidin 2 2D crystals

**Timing: <10 min**

Immobilization of lipid nanorods can be achieved by applying the lipid nanorod solution to the tamavidin 2 2D crystals.

24. Mix 9 μL of buffer solution (e.g., 20 mM HEPES-NaOH containing 150 mM NaCl, pH 7.0) and 1 μL of lipid solution (kept on ice after sonication) to prepare 0.1 mM of lipid solution.
25. Exchange the buffer solution on tamavidin 2 2D crystals to 0.1 mM of lipid solution.
26. Keep a drop of 0.1 mM of lipid solution on mica for 5 min.
27. Set up HS-AFM and start imaging.

**Figure 5:**
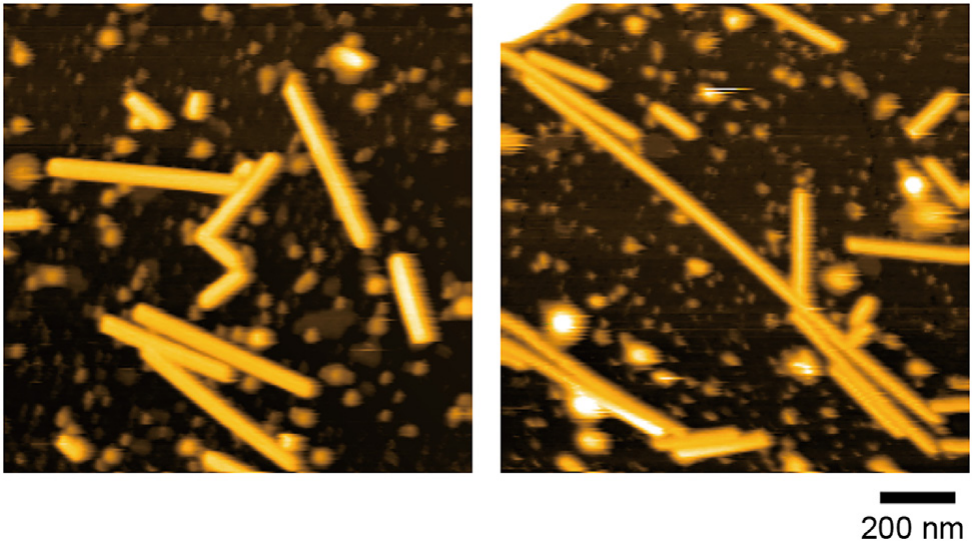
HS-AFM images of lipid nanorods immobilized on tamavidin 2 2D crystals The sizes of lipid nanorods are 30–50 nm in diameter and ∼0.1–1 μm in length. Scan range: 1250 nm × 1250 nm (250 × 250 pixels); Height scale: 0–45 nm (left) and 0–48 nm (right). Images were acquired at 1.5 s/frame.

***Note:***Figure 5 shows an example of the expected results. Typically 3–10 lipid nanotubes with different length (∼0.1–1 μm) are observed in 1 μm × 1 μm area.

28. Apply proteins of interest to the lipid nanorods immobilized on tamavidin 2 2D crystals after removing the glass stage from the HS-AFM setup or add proteins of interest to the imaging buffer while HS-AFM imaging.

***Note:*** You can incubate the proteins with lipid nanorods before applying it to tamavidin 2 2D crystals. The timing of addition of the proteins will depend on the proteins and the events you want to observe. For example, if you attempt to observe dynamin-coated lipid nanotubes, it would be preferable to mix dynamin and nanorods before fixation on tamavidin 2 2D crystals because fixation may interfere with the formation of dynamin assembly around the nanorods.^4^

## Expected outcomes

Immobilized lipid nanorods on tamavidin 2 2D crystals are the expected outcomes from this protocol (Figure 5). The lipid nanorods could be useful, especially for observation of proteins sensing membrane curvature such as proteins with BAR (Bin/Amphiphysin/Rvs-homology) domains.^8^ Nanorods are rigid enough to tolerate tapping force of the HS-AFM imaging. This protocol is an easy method to prepare immobilized lipid rod structures for HS-AFM observation once sufficient amounts of purified tamavidin 2 proteins are obtained.

## Limitations

The lipid nanorods formed by this protocol are rigid compared to those without galactosylceramide. Therefore, the dynamic processes of membranes, such as membrane fission, may be difficult to be observed.^4^ In addition, not only nanorods but also vesicular structures or layer structures could be observed on tamavidin 2 2D structures, as shown in Figure 5. You may need to select the region where nanorods with the appropriate length for your experiment are separated from other lipid assemblies.

## Troubleshooting

### Problem 1

It is important to keep the mica surface wet to prevent binding of contaminants throughout the experiment after deposition of the tamavidin 2 solution on the mica (Step 20). However, it is difficult to exchange buffers (wash) with keeping the surface wet.

### Potential solution

**Figure 6:**
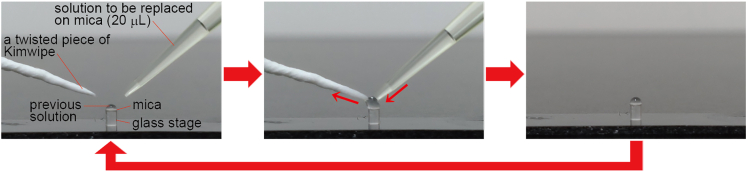
How to exchange buffer solutions on mica 20 μL of the solution to be replaced is pushed out from a micropipette (for 2–20 μL volume) on mica and absorbed with a twisted piece of Kimwipe simultaneously to keep the surface wet. It would be preferable to repeat this at least twice (≥2 × 20 μL).

Removing the previous solution on mica should be done at the same time as the addition of solution to be replaced (Figure 6). Because larger micropipettes are difficult to push out solutions little by little, use of micropipettes for 2–20 μL volume is recommended.

### Problem 2

Tamavidin 2 2D crystals were not formed on mica surface (Step 22).

### Potential solution

**Figure 7:**
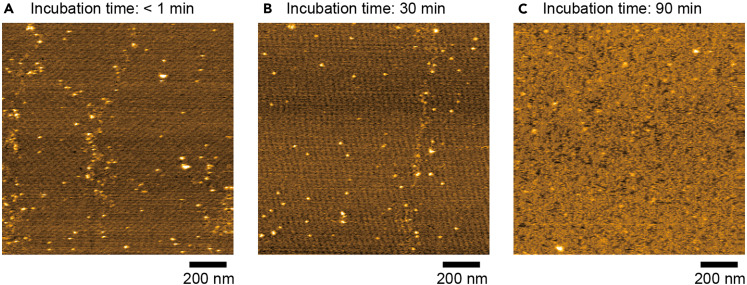
HS-AFM images of tamavidin 2 2D crystals of different incubation time (A–C) While incubation time ≤30 min produces the 2D crystals with almost no gaps (A, B), incubation time of 90 min causes fragmentation of the 2D crystals (C). Tamavidin 2 concentration after dilution by crystal buffer: 7.1 μM (A-C); Scan range: 1250 nm × 1250 nm (250 × 250 pixels) (A–C); Height scale: 0–7 nm (A, B) and 0–12 nm (C). Images were acquired at 1.5 s/frame.

The cause of the failure may be too long incubation time after dilution with crystal buffer (Step 18). Incubation time >60 min results in fragmentation of the 2D crystals as shown in Figure 7. Diluted tamavidin 2 solution should be applied on mica within 30 min. After 5 min incubation on mica, wash with 0.01% glutaraldehyde diluted by fixation buffer.

### Problem 3

When tamavidin 2 2D crystals were observed by HS-AFM, there were many aggregates or large defects (Step 22).

### Potential solution

**Figure 8:**
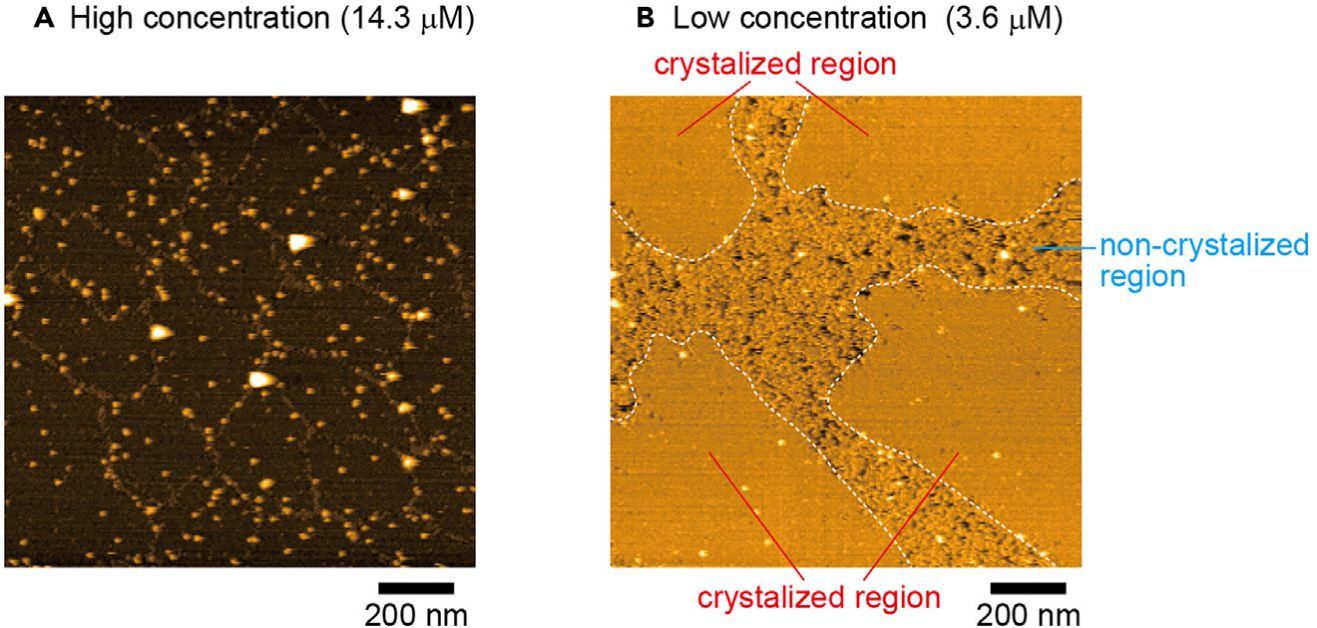
HS-AFM images of tamavidin 2 2D crystals of different tamavidin 2 concentration (A and B) Tamavidin 2 concentration after dilution by crystal buffer: 14.3 μM (A) and 3.6 μM (B); Scan range: 1250 nm × 1250 nm (250 × 250 pixels) (A, B); Height scale: 0–12 nm (A) and 0–10 nm (B). Images were acquired at 1.5 s/frame.

This would be caused by inappropriate tamavidin 2 concentration (Figure 8). Too high concentration results in many aggregates on 2D crystals (Figure 8A) and too low concentration produces non-crystalized region (Figure 8B). Reduce or increase protein concentration gradually by 20%.

### Problem 4

When streptavidin was used for tamavidin 2 as a biotin-binding protein and followed the same protocol described in ‘prepare tamavidin 2 2D crystals on mica’ subsection, no streptavidin 2D crystals on mica surface were confirmed by HS-AFM (Step 18–22).

### Potential solution

Streptavidin cannot be a substitute for tamavidin 2 because streptavidin is difficult to bind directly to the mica surface. The solid support (e.g., mica) must be previously covered by biotinylated lipid bilayers for preparing streptavidin 2D crystals. See the report by Yamamoto D et al.^9^ for the details about streptavidin 2D crystals.

### Problem 5

After lipid nanorods immobilization on tamavidin 2 2D crystals, many vesicular structures and planar lipid structures were observed by HS-AFM (Step 27).

### Potential solution

**Figure 9:**
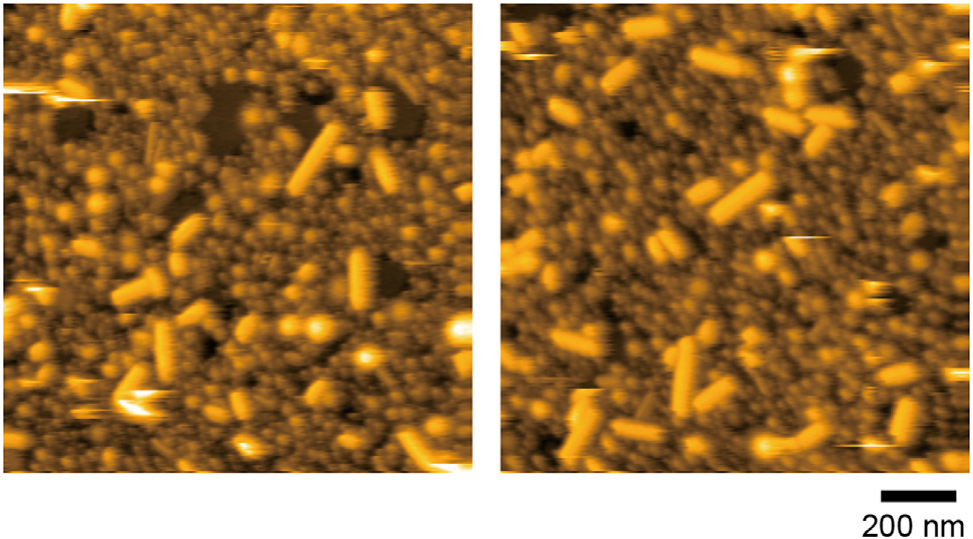
HS-AFM images of lipid nanorods sonicated by a tip sonicator for 60 s Much more vesicular structures are observed compared to Figure 5. Scan range: 1250 nm × 1250 nm (250 × 250 pixels); Height scale: 0–45 nm. Images were acquired at 1.5 s/frame.

Sonication of lipid mixture solution by tip sonicator for long time produces vesicular structures rather than nanorods. Figure 9 shows HS-AFM images of lipid nanorods sonicated by a tip sonicator for 60 s after bath sonication. Vesicular structures bound to the tamavidin 2 2D crystals can change to planar structures. Basically, tip sonication ≤5 s would be sufficient.

## Resource availability

### Lead contact

Further inquiries and requests for materials should be directed to the lead contact, Nobuo N Noda (nn@igm.hokudai.ac.jp).

### Materials availability

All requests for resources and reagents are available from the lead contact, subject to a Materials Transfer Agreement.

## Data Availability

This paper does not report any original code.
